# A mixed methods investigation of the relationship between blood donor policy, interest in donation, and willingness to donate among gay, bisexual, and other men who have sex with men in Ontario, Canada

**DOI:** 10.1186/s12889-022-13229-2

**Published:** 2022-04-28

**Authors:** JP Armstrong, David J. Brennan, David Collict, Maya Kesler, Tsegaye Bekele, Rusty Souleymanov, Daniel Grace, Nathan J. Lachowsky, Trevor A. Hart, Barry D. Adam

**Affiliations:** 1 Department of Sociology, York University, Vari Hall, Room 2060, 4700 Keele Street, Toronto, ON M3J 1P3 Canada; 2 Factor-Inwentash Faculty of Social Work, University of Toronto, 246 Bloor Street West, Toronto, ON M5S 1V4 Canada; 3 Ontario Institute for Studies in Education, University of Toronto, 252 Bloor Street West, Toronto, ON M5S 1V6 Canada; 4 Ontario HIV Treatment Network, Suite 600, 1300 Yonge Street, Toronto, ON M4T 1X3 Canada; 5 Faculty of Social Work, University of Manitoba (Fort Garry Campus), Room 521 Tier Building, 173 Dafoe Road West, Winnipeg, MB R3T 2N2 Canada; 6 Dalla Lana School of Public Health, University of Toronto, 155 College Street, Toronto, ON M5T 3M7 Canada; 7 School of Public Health & Social Policy, Faculty of Human & Social Development, University of Victoria, STN CSC, P.O. Box 1700, Victoria, BC V8W 2Y2 Canada; 8 Department of Psychology, HIV Prevention Lab, Ryerson University, 350 Victoria St, Toronto, ON M5B 2K3 Canada; 9 Department of Sociology, Anthropology, and Criminology, University of Windsor, 401 Sunset Ave, Windsor, ON N9B 3P4 Canada

**Keywords:** Gay, bisexual, and other men who have sex with men, Blood donation, Policy, Mixed methods, Canada

## Abstract

**Background:**

As of 2019, men who have sex with men (MSM) in Canada are ineligible to donate blood if they have had oral or anal sex with another man in the last 3 months. Deferral policies targeting MSM are largely interpreted as unjust by gay, bisexual, and other men who have sex with men (GBMSM) – shaping their desire to donate blood and engage with blood operators. This mixed methods study explores interest in blood donation among GBMSM as well as willingness (and eligibility) to donate under four different deferral policies.

**Methods:**

We surveyed 447 GBMSM who were recruited from the Ontario-wide #iCruise study. Participants were asked whether they were interested in blood donation and if they were willing to donate under each of our four deferral policies. We also completed interviews with 31 of these GBMSM. Participants were asked to describe their feelings about blood donation, their views on our different deferral policies, the impact of a policy change, as well as other means of redress.

**Results:**

Most participants (69%) indicated that they were interested in donating blood. Despite this, an interpretation of the MSM deferral policy as discriminatory was common among all participants. Our mixed methods findings indicate that, among those who were interested in blood donation, the adoption of one of the alternative policies presented in this study (specifically Policy 2 or Policy 3) would significantly increase the number of participants willing to donate and be viewed as “*a step in the right direction.*” However, many participants who were not interested in blood donation argued that a gender-neutral deferral policy would need to be implemented for them to donate. Participants recommended that blood operators consider efforts to repair relations with GBMSM beyond policy change, including pop-up clinics in predominantly queer areas and diversity sensitivity training for staff.

**Conclusion:**

We argue that the most impactful policy shift would be the implementation of an individual risk-based deferral policy that is applied to all donors regardless of sexual orientation or gender identity. However, given MSM’s historical exclusion from blood donations, blood operators should pair this policy shift with community relationship-building efforts.

## Background

In Canada, men who wish to donate blood are asked if they have had sex with a man in the last three months [1]. More specifically, the current policy excludes men who have sex with men (MSM) who have had anal or oral sex with another man in the past 3 months from donating blood. Health Canada regulations, such as the longstanding (yet significantly modified) deferral policy excluding MSM from blood donation, are argued to be justified as means of protecting the national blood supply and blood recipients. The original policy, deferring MSM indefinitely, was introduced in 1983 as a precautionary measure in response to the AIDS epidemic and the tainted-blood scandal, in which numerous Canadians contracted HIV through blood transfusion [2]. Since taking over management of Canada’s blood system from the Canadian Red Cross in 1998, Canadian Blood Services (CBS) and the province of Quebec’s blood supply agency, Héma-Québec, state that under their purview “there has not been a single recorded instance of blood-borne infection from either hepatitis C or HIV” in Canada [3]. However, evidence suggests that the decrease in HIV-positive donor rates since the 1990s, across many countries including Canada, is not a direct effect of any change in criteria, but likely due to enhanced public education about HIV risk factors, increased availability of HIV testing, and reduced stigma concerning gay, bisexual and other men who have sex with men (GBMSM) and HIV [4, 5]. HIV testing is “highly accurate and sexual preference is not synonymous with risk status” [6]. Blood operators in many countries, including Canada, have implemented nucleic acid testing (NAT) in addition to antibody testing for HIV, reducing the window period to less than 10 days, and have introduced automation and standardization of procedures resulting in extremely low error rates in testing and quarantining of positive units [4]. The only exception is for those who are taking HIV pre/post-exposure prophylaxis (PrEP/PEP), which include medications taken at and after potential exposure to prevent HIV. These medications can delay the detection of HIV [1].

Blood donation is “strongly marketed as an act of public altruism” [7] in Canada. GBMSM are interested in donating because of the social value, community benefits, and personal gratification placed on blood donation [8]. The Canadian national Sex Now Survey indicated that 92% of GBMSM were interested in donating blood, if eligible [9]. Grace et al. [10] interviewed HIV-negative GBMSM in Canada about their willingness to donate blood. The GBMSM who were interested in donating blood said they would gain personal satisfaction and civic pride from donating [10]. Further, many participants thought that their sexual and HIV prevention practices, along with a sense of “healthiness”, made them safe donors who should be eligible to donate blood [10]. But interest in donating blood and the ability to donate blood remain at odds for GBMSM, due to the MSM deferral policy. Participants in the Grace et al. [10] study most frequently attributed a lack of interest in donating blood to frustration over past and ongoing policies targeting MSM. They saw these policies as heterosexist and discriminatory because they exclude sexually active MSM from donating blood, leading to feelings of shame and marginalization. Indeed, GBMSM take issue with deferral policies that prohibit sex for any length of time [11]. Since the existing time-based deferrals are interpreted as discriminatory, many GBMSM favor a policy based on individual-level behavioral risk — a policy that is “gender neutral” — and do not consider a 3-month deferral to be a significant improvement from the previous 12-month deferral given that it seems discriminatory and out of step with scientific evidence [5, 12]. Zahner [13] refers to the current 3-month deferral as an excessive and needless buffer. GBMSM argue that sexual abstention, specifically between men, should not be a requirement that determines eligibility for blood donation [5, 10, 12].

In this mixed methods paper, we seek a better understating of interest in blood donation and willingness to donate among GBMSM in Ontario, Canada. More specifically, we investigate the potential impact a shift in policy might have on the interest as well as willingness (and eligibility) of these GBMSM. Further, we ask participants about modes of redress, beyond a change to the MSM deferral policy, that blood operators like CBS can employ to mend their damaged relationships with the GBMSM community. We employ the term MSM in reference to the deferral policy, which defers on the basis of sexual behaviour rather than sexual identity. However, though we recognize the policy relevance of this epidemiological category, it is not a sexual identity that our participant base used to describe themselves. When referring to our sample, we use the term GBMSM to signal the diverse sexual identities of our participant base. Further, the label GBMSM is more pertinent for understanding our participants’ attitudes concerning blood donation. Beliefs that the deferral policy is discriminatory are a consequence of the deferral’s stigmatization of GBMSM communities and identities via their exclusion from the donor pool [10].

## Methods

### Study background, design and data collection

This study recruited its participant base from the ongoing Ontario-wide mixed methods #iCruise study. The #iCruise study objective was to examine GBMSM’s sexual health outreach experiences through online services and mobile apps among. Participants for the #iCruise study were recruited via advertising on websites, mobile-apps, social media, and community-based organizations email listservs, between July 2017 and January 2018. Eligibility for the #iCruise study included participants who identified as male (cisgender or transgender); were 14 years or older; had ‘any’ sex with a man in previous year or were sexually/romantically attracted to other men or identified as gay, bisexual, queer or Two-Spirit; and lived or worked in Ontario or visited Ontario four or more times in the year prior. Data collection for the #iCruise study was completed online. Detailed methods have been previously described [14].

Following the completion of the second #iCruise questionnaire, participants (deemed eligible for this study) were asked if they were interested in participating in a study on blood donation. Participants who had completed both of the #iCruise questionnaires; self-reported as HIV-negative or HIV status unknown; had provided a forward sorting address (first three letters/numbers of their postal code) or city of residence; and were 17 years or older (able to donate blood in Canada) were eligible for this study. Eligible #iCruise participants who agreed to be a part of this study were sent the questionnaire 3 months after completing the final #iCruise questionnaire. This study’s questionnaire was completed by 447 GBMSM between April 2018 and June 2018. Participants completed an online questionnaire covering six domains: 1) demographic information, 2) HIV status, STI status, substance use, 3) sexual behaviors (over a three-month period), 4) experiences with Canadian Blood Services, 5) knowledge of the MSM deferral policy and 6) willingness to donate under various deferral policies. Survey participants were compensated $15 CAD for completing the questionnaire.

After the study questionnaires were completed, 31 hour-long interviews were conducted in 2018 and 2019. Interview participants were purposively recruited from the pool of survey participants. The following sociodemographic characteristics, collected during the quantitative arm of the study, guided this purposive sampling: 1) interest in blood donation, 2) sexual orientation, 3) ethnicity, and 4) rurality. This sampling approach was employed as a means of balancing these sociodemographic characteristics among our pool of interview participants. Further, it ensured an adequate sampling of participants who indicated an interest in blood donation as well as those who did not, providing us with enough data to gain in-depth qualitative insight into both interest and disinterest. Interviews were conducted either in-person (at the University of Toronto) or over the phone using a semi-structured guide with additional scripted probes, providing an in-depth exploration of experiences and perspectives of blood donation policies and practices. Interview participants were offered $30 CAD for their time. All methods were carried out in accordance with relevant guidelines and regulations.

### Quantitative measures

Age of participants was categorized into: 17–29, 30–49, and 50 and older years of age. Sexual orientation was dichotomized into gay vs. bisexual/other (other includes Two-Spirit, mostly straight, queer, asexual, pan-sexual, questioning, and unsure). Ethnoracial identity was categorized into seven categories (White, African/Caribbean/Black, East Asian/South East Asian, South Asian, Indigenous, Latino/Brazilian/South American, or other) and collapsed further into a binary variable (White vs. non-White) due to small sample sizes. We categorized relationship status into married/living with a common-law partner vs. single/polyamorous/divorced/separated/widowed. We also collected data on highest level of education (high school completion or lower, some post-secondary education, or university completion or higher). Employment status was categorized into a binary variable (working full-time/part-time vs. unemployed/retired/student) and we categorized annual personal income (in CAD) into four categories (less than $20,000, $20,000 to $39,999, $40,000 to $59,999, or $60,000 or greater). Participants were also asked whether anyone was aware of their sexual orientation (yes vs. no) and if they lived ‘in a rural or remote area of the province’ (yes vs. no). We assessed participants’ interest in blood donation with a single question: “*Have you ever been interested in donating blood in Canada*?” Based on responses to this question, we classified participants into two groups (yes vs. no/don’t know/prefer not to answer).

Participants were presented with four policy options (Table 1) and asked if they would be willing to donate under each separate policy, regardless of eligibility. Policy 1 excludes MSM from donating if they have had anal or oral sex with another man in the last 3 months. At the time of data collection, a variant of this policy with a 12-month deferral was in place in Canada and Policy 1 was presented to participants as a hypothetical. However, a deferral policy identical to Policy 1 was implemented by CBS in 2019 [1]. We also presented three hypothetical ‘alternative policies’ that altered the sexual behaviours affecting eligibility. The development of these three alternative policies was guided by recent epidemiological research as well as pertinent CBS eligibility requirements. The risk of HIV transmission during oral sex is considerably lower than the risk from anal sex; furthermore, the risk of HIV acquisition varies widely depending on the self-reported HIV-status of partners and consistent condom use [15–22]. The three alternative policies maintained the 3-month deferral but altered the sexual behaviours affecting eligibility based on these epidemiological findings. Each is less restrictive than the last. Policy 2 defers men who have anal sex with another man but allows for oral sex with another man. Policy 3 permits anal sex with another man as long as condoms were used 100% of the time. We did not present a similar alternative deferral policy, including the consistent use of PrEP as a prevention strategy, because individuals who have taken this medication within 4 months of screening are deferred due to the medication’s potential to delay detection of HIV [1, 23]. Finally, Policy 4 permits anal sex with another man regardless of condom use. In Canada there is a gender-neutral 12-month deferral for any individual who has had sex with an HIV-positive partner [23, 24]. As such, Policy 3 and 4 include the stipulation that sexual partners must be HIV-negative (thus excluding those who have had anal sex with an HIV-positive partner). We also assessed participants’ eligibility to donate blood under the four policies described above using self-reported data on participants’ sexual behaviors across a 3-month period (reported in the questionnaire). If participants reported anal sex (receptive or insertive), they were asked about the HIV status of their partners and frequency of condom use.

**Table 1:** Deferral policies

Screening policy	Eligibility criteria (all with a 3-month deferral)
Policy 1	MSM are ineligible to donate if they have had oral or anal sex with another man
Policy 2	MSM are ineligible to donate if they have had anal sex with another man (irrespective of oral sex)
Policy 3	MSM are eligible to donate if they have had anal sex as long as it was with an HIV-negative partner using condoms 100% of the time (irrespective of oral sex)
Policy 4	MSM are eligible to donate if they have had anal sex as long as it was with an HIV-negative partner (irrespective of oral sex and irrespective of condom use)

### Statistical analyses

We summarized sociodemographic characteristics of the study sample using descriptive statistics; frequencies for categorical variables and median and inter-quartile range for continuous variables. Associations between sociodemographic characteristics and interest in blood donation were tested using chi-square tests and Fisher’s exact tests. We compared the overall proportion of participants willing and eligible to donate blood under Policy 1 with the proportion of participants willing and eligible to donate under the other three policy options. We also compared the number of participants willing to donate under Policy 1 with the number of participants willing to donate under the other three policies stratified by interest in blood donation. We used McNemar chi-square tests to test the statistical significance of these differences. Reported *p*-values were from two-sided tests and determined at the *p* < 0.05 level. All statistical analyses were conducted using SAS software version 9.4.

### Qualitative analysis

The first stage of our grounded analytic approach involved developing a codebook inductively from the raw data [25] and applying these codes to the dataset. An initial reading of a subset of the transcripts (purposively selected to reflect the demographics of our overall interview cohort) was completed by four team members. Each team member read 3–5 different transcripts and took note of significant concepts. After reviewing our notes and combining overlapping concepts into single codes, we produced a single codebook which reflected all our readings of the data. Following this, one team member uploaded the codebook into NVivo software version 10 and analyzed all 31 transcripts – applying the codes developed from the collective reading. During the second stage of our analysis, we brought together interrelated codes and reread the associated data as a means of interrogating the relationship between these concepts and achieving a higher level of abstraction. From this two-stage grounded analysis we were able to identify recurrent or common themes.

## Results

### Interest in blood donation

Four hundred and forty-seven GBMSM completed the CBS questionnaire as part of the broader #iCruise study (Table 2). Most of the participants were younger than 50 years of age (82%), identified as gay (81%) and White (62%), were neither married or common-law (83%), worked full/part time (78%), had a personal income of $20,000 or more (70%), ‘out’ about their sexual orientation to at least one person (96%), and did not live in a rural or remote area of the province (86%). Sixty-nine percent (*n* = 309) of GBMSM who participated in this study reported interest in donating blood in Canada. Interest differed significantly by age – participants between 30 and 49 demonstrated significantly less interest than participants who were 17 to 29 years or 50 years or older (*p* = 0.038). Interest in blood donation did not differ significantly (*p* > 0.05) by any of the other demographic variables presented in Table 2.

**Table 2:** Demographic characteristics of study participants by interest in donating blood

Demographic characteristics	All participants							Interview participants						
	Have you ever been interested in donating blood in Canada?							Have you ever been interested in donating blood in Canada?						
	Yes		No/don’t remember/prefer not to answer		Total sample		*p*-value	Yes		No/don’t remember/prefer not to answer		Total sample		***p*-value**
	[*n* = 309]		[*n* = 138]		[*N* = 447]			[*n* = 21]		[*n* = 10]		[*n* = 31]		
Age							0.038							^**b**^
17–29	147	(48%)	57	(41%)	204	(46%)		11	(52%)	^a^	^a^	15	(48%)	
30–49	99	(32%)	61	(44%)	160	(36%)		7	(33%)	^a^	^a^	10	(32%)	
≥ 50	63	(20%)	20	(14%)	83	(19%)		^a^	^a^	^a^	^a^	6	(19%)	
Sexual orientation							0.631							0.429
Gay	247	(80%)	113	(82%)	360	(81%)		13	(62%)	8	(80%)	21	(68%)	
Bisexual/other	62	(20%)	25	(18%)	87	(19%)		8	(38%)	^a^	^a^	10	(32%)	
Ethnicity							0.273							0.458
White	196	(63%)	80	(58%)	276	(62%)		12	(57%)	^a^	^a^	16	(52%)	
Non-White	113	(37%)	58	(42%)	171	(38%)		9	(43%)	6	(60%)	15	(48%)	
* African/Caribbean/Black*	*24*	*(8%)*	*10*	*(7%)*	*34*	(*8*%)		^a^	^a^	^a^	^a^	^a^	^a^	
* East Asian/South East Asian*	*24*	*(8%)*	*26*	*(19%)*	*50*	(*11*%)		^a^	^a^	^a^	^a^	^a^	^a^	
* South Asian*	*16*	*(5%)*	^a^	^a^	*21*	(*5*%)		^a^	^a^			^a^	^a^	
*Indigenous*	*17*	*(6%)*	^a^	^a^	*22*	(*5*%)		^a^	^a^	^a^	^a^	^a^	^a^	
*Latino/Brazilian/South American*	*20*	*(6%)*	*9*	*(7%)*	*29*	(*6*%)		^a^	^a^	^a^	^a^	^a^	^a^	
*Other*	*12*	*(4%)*	^a^	^a^	*15*	(*3*%)								
Marital status							0.690							0.906
Married/common-law partner	54	(17%)	22	(16%)	76	(17%)		^a^	^a^	^a^	^a^	^a^	^a^	
Single/polyamorous/ divorced/separated/widowed	255	(83%)	116	(84%)	371	(83%)		17	(81%)	9	(90%)	26	(84%)	
Education level							0.746							^b^
≤ High school	29	(9%)	13	(9%)	42	(9%)		^a^	^a^			^a^	^a^	
Some post-secondary education	132	(43%)	54	(39%)	186	(42%)		10	(48%)	7	(70%)	17	(55%)	
University degree or higher	147	(48%)	71	(51%)	218	(49%)		9	(43%)	^a^	^a^	13	(12%)	
Working FT/PT							0.274							0.273
Yes	245	(79%)	103	(75%)	348	(78%)		^a^	^a^	^a^	^a^	7	(23%)	
No	64	(21%)	35	(25%)	99	(22%)		17	(81%)	7	(70%)	24	(77%)	
Personal income							0.780							^b^
< $20,000	76	(25%)	39	(28%)	115	(26%)		9	(43%)	^a^	^a^	13	(42%)	
$20,000—$39,999	78	(25%)	33	(24%)	111	(26%)		^a^	^a^	^a^	^a^	^a^	^a^	
$40,000—$59,999	68	(22%)	26	(19%)	94	(21%)		^a^	^a^	^a^	^a^	6	(19%)	
≥ 60,000	72	(23%)	34	(25%)	106	(24%)		^a^	^a^	^a^	^a^	7	(23%)	
Not reported	15	(5%)	6	(4%)	21	(5%)		^a^	^a^	^a^	^a^	^a^	^a^	
Is anybody aware of your sexual orientation?							0.112							0.999
Yes	299	(97%)	129	(93%)	428	(96%)		18	(86%)	9	(90%)	27	(87%)	
No/prefer not to answer	10	(3%)	9	(7%)	19	(4%)		^a^	^a^	^a^	^a^	^a^	^a^	
Live in rural/remote area							0.804							0.429
Yes	43	(14%)	18	(13%)	61	(14%)		8	(38%)	^a^	^a^	10	(32%)	
No	266	(86%)	120	(87%)	386	(86%)		13	(62%)	8	(80%)	21	(68%)	

^a^Data suppressed due to small cell size

^b^Statistical tests were not performed due to small cell sizes

Qualitative findings provided insight into the lack of interest in blood donation indicated by almost 31% (*n* = 138) of the survey participants. The existence of a “discriminatory” policy singling out MSM was a common reason for interviewees’ disinterest in blood donation. As one participant (not interested in donating blood, 30–49) bluntly stated: “*It’s* [the MSM deferral policy] *homophobic of course, I think it’s homophobic and discriminatory*.” Another participant (not interested in donating blood, 17–29) expanded on the “discriminatory” nature of the deferral: “*I don’t think they would ask a heterosexual male or a heterosexual female if they’ve had sex at all… I think it’s just very unfair. Why am I being judged for having sex with another man?*” Indeed, regardless of interest in blood donation, a significant number of participants suggested that the deferral policy unfairly targets gay and bisexual men in a disproportionate manner compared with the broader population. A participant (interested in donating blood, 17–29) who recognized the importance of donating blood stated: *“I think the fact that these policies have been in place has kind of turned me off* [to] *the idea of donating blood, even though I know it’s a really important thing to do that can save a lot of lives. It sort of feels to me like they’ve put up this big sign that says they don’t want my blood.*” One participant (interested in donating blood, 30–49) described their lack of willingness to donate under an MSM deferral policy as an act of protest: *“I’m not going to help out, you’re not going to get my blood, and it’s my way of saying it’s wrong. I wouldn’t want to do anything that justifies this kind of policy.”* Another participant (interested in donating blood, 30–49) described the deferral policy as homophobic, arguing that the continued use of a deferral policy for MSM was rooted in systemic homophobic beliefs: “*I think that we have this paranoid, almost homophobic culture, and sex-negative culture that actively refuses the idea of queer and men who have sex with men as part of everyday life. I think they think of it as always contaminated, and incapable of being anything but contaminated, and a fear of that contamination.*” Participants noted that the deferral policy was targeted directly towards MSM via the assumption that gay men are “careless” in their sexual risk-taking behaviours. One participant (interested in donating blood, 17–29) made clear the relationship between deferral policies for MSM and stereotypical beliefs about GBMSM dating to the AIDS epidemic and the tainted-blood scandal: “*When the policies were made, I believe that they believed that every single homosexual male could be HIV-positive, and there was such a stigma around it* [HIV] *at the time.”*

As was the case with 69% (*n* = 309) of survey participants, overwhelmingly interviewees suggested they were interested in donating. Participants described blood donation as an altruistic behavior and a means of *“helping someone in need,”* with one participant also citing the Canadian Blood Service’s own slogan for donation: *“it’s* [blood] *in you to give.”* Indeed, one participant (interested in donating blood, 30–49) noted a desire for blood donation as a form of broader public and community service: *“I just think it’s the ethical thing to do. I think… for the same reason why I’d like to sign my organ donor card, or why I give money to charity, or why … it just seems morally right. You help people if you can help them.”*

Among the participants interested in donation, the vast majority noted that they would only donate if they happened to be eligible, making it clear that they would not alter their sex practices in order to meet donor eligibility criteria. One participant (interested in donating blood, 17–29) summarized this theme, asking: *“why would I ever put myself at personal loss of sexual intimacy for a period of time in order to be able to donate to them?”* Participants spoke about engaging in sex with other men but cited precautions such as utilizing condoms, PrEP, and frequent testing as sufficient means of ensuring their personal sexual safety. Indeed, many participants viewed their sex practices as safe and taken with precaution to avoid sexual risks. A small number of participants even indicated that they have self-assessed their blood as “safe” and lied during the screening process as a means of donating blood. One participant (interested in donating blood, 17–29) stated: *“I know that I’m being safe, and I know that my blood is safe to donate… It shouldn’t really be an issue who I am having sex with.”* When asked to elaborate on their noncompliance, they continued: “*I know that my blood will not be donated otherwise, or that I know that my blood will be not used, which is frustrating*.” This suggests a disjuncture between the definition of sexual risk guiding the deferral of MSM and how participants interpreted their own risk; in other words, participants saw the blanket deferral of sexually active MSM as outdated rather than rooted in a contemporary, evidence-based understanding of HIV transmission and prevention. Participants argued for a “systemic change” aimed at bringing the deferral policy in line with the current scientific knowledge as well as increasing eligibility among GBMSM.

### Willingness and eligibility under deferral policies

The number of participants willing and eligible to donate blood under the four potential deferral policies are reported in Table 3. Less than half (42.3%) of GBMSM were willing to donate under Policy 1. A significantly (*p* < 0.05) higher number of participants were willing to donate when comparing Policy 2 and Policy 3 (but not Policy 4) to Policy 1. Moving across the four policies, as the sexual behavior requirements became less strict, the number of participants eligible to donate increased. The number of participants who were eligible to donate was significantly (*p* < 0.05) higher under Policy 2, Policy 3 and Policy 4 when compared to Policy 1, with up to 80% of GBMSM eligible to donate under the Policy 4. Overall, only 12.3% of GBMSM were both willing and eligible to donate under Policy 1. The number of participants willing and eligible to donate was significantly (*p* < 0.05) higher under the subsequent policies, reaching its highest point (36.0%) under Policy 4.

**Table 3:** Willingness and eligibility to donate blood among study participants by interest in donating blood

Screening policy	Willing			Eligible	Willing and eligible
	**Ever interested in donating blood?**		**Total sample (*N* = 447)**	**Total sample** **(*N* = 447)**	**Total sample** **(*N* = 447)**
	**Yes (*n* = 309)**	**No/don’t remember/ prefer not to answer (*n* = 138)**			
	n (%)	n (%)	n (%)	n (%)	n (%)
Policy 1	147 (47.6)	42 (30.4)	189 (42.3) ^b^	131 (29.3)	55 (12.3)
Policy 2	165 (53.4) ^c^	39 (28.3)	204 (45.6) ^a b^	183 (40.9) ^a^	78 (17.5) ^a^
Policy 3	178 (57.6) ^c^	49 (35.5)	227 (50.8) ^a b^	219 (48.9) ^a^	105 (23.5) ^a^
Policy 4	157 (50.8)	44 (31.9)	201 (45.0) ^b^	357 (79.9) ^a^	161 (36.0) ^a^

^a^Significantly (*p* < 0.05) higher than Policy 1 among the total sample

^b^Difference between those interested and not interested in donating is significant (*p* < 0.05) within each screening policy

^c^Significantly (*p* < 0.05) higher than Policy 1 among those interested in donating

When compared with participants not interested in blood donation, participants interested in blood donation were significantly (*p* < 0.05) more likely to indicate they were willing to donate under all four policy options (Table 3). Further, among those interested in donating, the numbers of participants willing to donate under Policy 2 and Policy 3 were significantly (*p* < 0.05) higher than the number of participants willing to donate under Policy 1. Notably, this was not true among participants not interested in donating blood – the number of participants willing to donate did not vary significantly (*p* > 0.05) across the four policy options.

Interview participants were asked to provide their thoughts on the various components of the four deferral policies as a means of better understanding why willingness might differ between said policies. Participants overwhelmingly interpreted anal sex as a high-risk sexual behaviour. In fact, no participants stated a belief in anal sex as a low-risk sexual behaviour. Participants expressed knowledge of anal sex as a primary means of transmitting HIV among GBMSM and other populations and that it is understandable that anal sex is included in the MSM deferral policy. However, many participants recognized oral sex as a low-risk sexual behaviour with a negligible risk of HIV transmission, challenging the definition of sex employed in the MSM deferral policy. One participant (interested in donating blood, 17–29) found the inclusion of oral sex to be comical: “*I’m trying not to laugh because there is such a low risk of oral transmission. There’s no reason to put oral in there*.” Participants often framed their interpretation of oral sex as a low-risk sexual behaviour in relation to their interpretation of anal sex as a high-risk sexual behaviour. A participant (not interested in donating blood, ≥ 50) stated: “*I think* [HIV transmission] *is much more possible with anal than it is with oral.”*

Interviewees generally agreed on consistent condom use as an effective prevention strategy for HIV transmission, one that should be taken into consideration during eligibility assessments. However, a small number of participants identified concerns regarding the inclusion of consistent condom use in the deferral policy. The effectiveness of condoms, namely the argument that condoms are “*not stopping everything,*” and uncertainty about whether or not a partner “*has* [always] *used a condom during a* [sexual] *encounter*” underscored these concerns. When discussing the inclusion of screening for consistent condom use, participants often referenced PrEP as an HIV prevention strategy, which suggests that these participants were unaware that individuals taking this medication are subject to deferral [1]. One participant (interested in donating blood, 17–29) argued: “*It* [the MSM deferral policy] *should really only reflect whether I used condoms or whether I’m taking PrEP or whether I’m taking the due diligence*.” However, when compared with beliefs about the effectiveness of consistent condom use, participants were more varied in their beliefs regarding whether PrEP should, or should not, be considered in a deferral policy as an effective prevention strategy. Some participants in favor of considering PrEP note that continued use requires frequent HIV-testing. On the other hand, a participant (interested in donating blood, 30–49) argued, *“From a scientific basis, it’s* [preventive benefit has] *been proven, but it’s still very new, and it relies on individuals to take PrEP every day. I don’t trust people to have that kind of commitment.”* Many participants did not approve of the consideration of “*unprotected anal sex*” (i.e. no condom use or inconsistent condom use) largely due to the interpretation of anal sex as high-risk as well as concerns about potential donors lacking concrete knowledge of their sexual partners’ HIV status – some participants noting that it is difficult to ever be certain of a sexual partner’s status. However, some participants noted that individuals in monogamous sexual partnerships should not be subject to any deferral of anal sex, irrespective of condom use. As suggested by one participant (not interested in donating blood, 17–29), “*I’ve been with one person for years and years and years…* [we] *should be able to give blood without having to wait*.”

### Impact of policy change and the need for other forms of redress

Many participants stated that a policy change would have a positive impact on their views of blood donation and CBS. A recurrent argument was that such a change would be symbolic of progress towards a deferral policy grounded in contemporary scientific knowledge of and technological advancements in HIV transmission and detection. Participants described such a change as “*a step in the right direction”* and implied that policy change would signal a shift away from what they considered to be a discriminatory practice rooted in misinformation. One participant (interested in donating blood, 17–29) described this, stating: “*I know that the fact that it* [the deferral] *was reduced to only 12 months* [from a 5-year deferral] *was heralded as a progressive move from the government, but it’s still not quite there yet.”* Participants also suggested that a policy change would increase their likelihood of donating blood in the future. A participant (not interested in donating blood, 17–29) noted: “*It would give me a little bit peace of mind. Like I would say it would probably persuade me to donate.”* Finally, some participants remarked that such a change would facilitate institutional change. One participant (interested in donating blood, 30–49) argued: “*I think that anything that eases or lessens institutional homophobia is something we should probably investigate, and I think it* [policy change] *would make that the case.”*

But some interview participants, predominantly those who were not interested in donating blood, stated that they were not convinced that a change to any of the listed policies would shift their beliefs regarding blood donation. These participants highlighted that anal sex is not practiced exclusively by GBMSM, arguing that the continued deferral of MSM specifically under our policies was “homophobic.” Similarly, participants emphasized that HIV knows no single orientation and suggested that everyone must be conscious of their sexual health. A participant (preferred not to answer question about interest, 17–29) argued “[HIV]*… doesn’t discriminate. Everyone can get it. You don’t practice safe sex you can get it.*” Many noted that they would continue to have a “negative” perception of blood donation so long as there was a deferral policy targeting MSM. One participant (not interested in donating blood, 30–49) summarized this: “*I have a somewhat negative opinion about it* [blood donation] *and would be unlikely to change that unless I believed that they were not discriminating against gay men*.” Common was the desire for a gender-neutral, individual risk-based policy. One participant (not interested in donating blood, ≥ 50) emphasized this, calling for a policy “*that stopped focusing on orientation*” and “*was the same policy for everyone*.”

Participants argued that CBS also needs to engage in community-building efforts with GBMSM. These efforts were perceived as necessary to demonstrate a commitment to structural change and the removal of discriminatory barriers. One participant (interested in donating blood, 17–29) stated: *“They need to blast out marketing and have images of guys walking into the donation clinic holding hands and language that says we want your blood no matter who you are. They’ve really got to have a PR* [public relations] *spin to try to repair their relationship to this community”.* Other participants spoke of a desire for CBS to hold pop-up clinics in predominantly queer areas. Participants also proposed that CBS have staff engage in diversity sensitivity training as a means of addressing concerns of discrimination in interactions with blood operators. Participants who advocated for sensitivity training believed that this training would alleviate potential donors’ fear of prejudicial treatment from CBS employees when disclosing personal information.

## Discussion

These findings offer significant insight into interest in blood donation among GBMSM as well as the impact of various policy changes on participants’ eligibility and willingness to donate blood. This study also identifies modes of community redress recommended by participants that blood operators might pursue as a means of healing their relationship with the GBMSM community. First, participants’ reasons for being interested in donating blood are generally consistent with other studies. Indeed, participants described willingness to donate as a means of civic pride and personal satisfaction/self-fulfillment [8, 10], and as an act they wish to engage in as a form of altruism and a broader public and community service [12]. Generally, participants interpreted their own sex practices as safe and low risk, particularly given their utilization of protective measures such as consistent condom use as well as frequent HIV testing. Though some participants identified PrEP as an effective risk-mitigator which should be considered when assessing donor eligibility, other participants noted some hesitation with the inclusion of PrEP use due to the perceived novelty of these interventions. Notably, none of the interview participants recognized that, in Canada, potential donors are screened for PrEP use and deferred if taking PrEP [1]. Other researchers have found that GBMSM are generally knowledgeable about HIV related risk and good at assessing their own risk level [10], partly because of a more than 30-year history of “safer sex training” by AIDS service organizations that highlighted oral sex as “low risk” for HIV transmission when compared to the “higher risk” associated with anal sex [26]. Yet, GBMSM’s interpretation of risky sexual behaviours differed largely from the sexual behaviours excluded under the MSM deferral policy. Many participants disagreed that oral sex is a high-risk sexual behaviour, particularly in relation to HIV transmission. Participants understood anal sex as a generally a high-risk sexual behaviour and understood why potential donors who have had anal sex are deferred. However, they felt this deferral should be applied to all donors — giving consideration to individual prevention strategies — rather than applied to MSM specifically.

Participants who were interested in donating blood made it clear that it is the responsibility of CBS to ensure their eligibility, noting the need to implement a policy that ensures the safety of the blood supply without rendering all sexually active MSM ineligible. Unsurprisingly, the number of participants eligible for blood donation increased when the inclusion criteria were eased. The number of participants eligible was highest under a policy that would allow an individual who has engaged in anal sex with an HIV-negative partner to donate, even if they have used a condom less than 100% of the time (Policy 4). Interestingly, the number of participants willing to donate was not highest under this policy. It is possible that, due to participants’ interpretation of anal sex as a high-risk sexual behaviour, participants may be less comfortable with this deferral policy. This lack of comfort might also be connected to participant’s apparent lack of faith in the reliability of knowledge of partners’ HIV status. Participants noted that it can be difficult to be certain of a partners’ HIV status, and thus difficult to self-report partner HIV status during screening. This problematizes the reliability of the presently employed gender-neutral screening question that ascertains whether or not a potential donor has had sex with an HIV-positive partner [23, 24]. Further, it may explain why participants state they are generally uncomfortable with a policy that allows anal sex without consistent condom use, preferring an individual risk-based policy that allows for anal sex as long as donors are following HIV prevention strategies, namely consistent condom use. But some participants highlight that even consistent condom use is not foolproof – citing similar reliability concerns regarding knowledge that a partner has used a condom as well as concerns regarding the effectiveness of condom use. Indeed, the effectiveness of consistent condom use as an HIV-prevention strategy is strong, yet partial [20]. There is a gap between perfect use failure rate and typical use failure rate, due in part to condom use errors and problems [27]. Despite this, the alternative policy allowing for anal sex incorporating consistent condom use was deemed most favourable by our participants, with 50.8% of GBMSM indicating a willingness to donate under this policy. An additional consideration suggested by some participants was that individuals in monogamous sexual partnerships be exempt from any deferral based on anal sex (irrespective of condom use).

This research distinguishes between general interest in blood donation and willingness to do so under specific policies finding that willingness to donate is mediated by interest in blood donation. Individuals who identified themselves as interested in blood donation were significantly more likely to be willing to donate (under all four policies presented in this study) when compared to GBMSM who were not interested in donating blood. There are numerous reasons for disinterest in blood donation, many which extend beyond GBMSM and affect the broader population. The most common reason is inconvenience [28, 29]. In addition, the donation process elicits fear in many, including fear of needles, sight of blood, and can lead to physical reactions such as fainting [29]. But our findings suggest that the potential impact of policy change may also be limited by GBMSM’s perception of the MSM deferral policy as unjust. Indeed, participants overwhelming interpreted the MSM deferral policy as discriminatory and one that disproportionately targets gay and bisexual men. This is consistent with other literature that finds similar sentiment among GBMSM [5, 6, 10, 12]. GBMSM understand blood donation policies as discriminatory, heterosexist, [6, 10] furthering social marginalization, [8] and out of line with scientific evidence [10]. This interpretation was salient among our participants, both those who identified themselves as interested in blood donation, as well as those who were not. Thus, for some participants, the desire to donate blood persists despite their interpretation of an MSM deferral policy as unjust. This is in line with past research suggesting that feelings of altruism and self-fulfillment elicited by blood donation outweigh most concerns of GBMSM already inclined to donate [10, 30]. Some of these participants (self-assessed as safe and otherwise eligible) also revealed that, in the past, they have lied about having sex with other men during screening as a means of donating blood. These results demonstrate that if the MSM deferral policy is interpreted as unjustly exclusionary and unscientific a consequence may be a loss of faith in and increased nonadherence to screening among GBMSM [31]. This poses problems for the broader screening process, which relies on the honesty of donors [3] and demonstrates the importance of implementing an evidence-based deferral policy.

A modification to the MSM deferral policy was interpreted as a step towards bringing it in line with significant advances to the understanding of HIV transmission as well as the evolving ability to detect HIV [32]. Some participants noted that a policy shift would be viewed as meaningful and might motivate them to donate. However, other participants (primarily those who were not interested in blood donation) were clear about their negative view of blood donation so long as there was any policy applied solely to MSM – including the policy alternatives presented in this study. Many of these participants, who saw themselves as engaged in low-risk sexual behaviours yet ineligible for having sex with other men, pointed out that any eligibility criteria that continues to allow others who are engaged in the same sexual behaviours (and in some cases higher-risk sexual behaviours) to donate blood merely because their partner is of the opposite gender [33] is unsatisfactory. This might explain why, among participants who indicated that they are not interested in donating blood, the number of GBMSM willing to donate did not increase significantly across our policies. Thus, if one of the alternative policies proposed here were implemented, it might not mobilize GBMSM who are already disinterested in donation due to their interpretation of an MSM deferral as discriminatory practice. When asked to describe an ideal policy, many noted a preference for an individual risk-based deferral policy that is applied to all donors regardless of sexual orientation or gender identity. Therefore, to remedy disinterest in blood donation among GBMSM, simply modifying the current policy applied to MSM may not be enough – the most impactful policy change could be a shift to a gender-neutral, individual risk-based deferral policy. Such a policy might be the only means of satisfying those who are disinterested in donating or unwilling to donate under any policy that singles out MSM. Further, participants frequently suggested specific means by which blood operators such as CBS can engage in community relationship-building, including engaging in PR campaigns and pop-up clinics in predominantly queer areas to intentionally interacting with and encouraging donation from GBMSM as well as requiring staff and volunteers to complete diversity sensitivity training to encourage positive future interactions with GBMSM and other queer donors. Such efforts, paired with the implementation of a gender-neutral, individual risk-based deferral policy, would likely be perceived as recognizing the history of institutionalized discrimination embedded in the MSM deferral policy and its impact on GBMSM’s interest and willingness to donate blood.

### Limitations

Interest in blood donation and willingness to donate under the four policies discussed in this paper were measured while a 12-month MSM deferral policy was still in place in Canada. We cannot speak to the effect of the deferral period’s reduction from 12 to 3 months on participants’ interest in blood donation or willingness to donate.

This study recruited participants from the #iCruise study. As a result, our participant base may be more sexually active than the broader GBMSM population due to the #iCruise eligibility criteria.

Policy 3 and Policy 4 allow anal sex but include the stipulation that sexual partners must be HIV-negative. The rationale for including this stipulation was that the Canadian eligibility criteria excludes any individual who has had sex with an HIV-positive partner within 12 months of screening [23, 24]. However, we recognize that these alternative policies can be read as falsely suggesting that anal sex (irrespective of condom use) with a partner who has an undetectable viral load is a high-risk behaviour. This is not the case [34], and we encourage future research to investigate the possibility of a donor policy allowing for sex with an HIV undetectable partner.

## Conclusions

The results of this study provide important considerations for future blood donation policy directions and implementation in Canada as well as in other countries reconsidering their deferral of MSM. Indeed, as of December 2021, Health Canada is considering CBS’ recommendation that Canada drop its MSM deferral policy and adopt a gender-neutral, individual risk-based deferral policy for all prospective blood donors [35]. Though the specifics of this recommendation have not yet been announced, CBS explicitly state that they are “on the same journey” as the United Kingdom [35]. The United Kingdom’s gender-neutral policy defers “anyone who has had anal sex with a new partner or multiple partners in the last three months*,”* allowing individuals in monogamous sexual partnerships to donate, irrespective of anal sex [36]. If CBS’ recommended policy is in line with the United Kingdom’s deferral policy, then the policy shift proposed in this paper generally supports this recommendation. The results of this paper also indicate GBMSM’s support for considering other HIV prevention strategies, namely consistent condom use, in the assessment of donor eligibility. However, concerns about the inclusion of consistent condom use highlighted by some of our participants suggest the need for a better understanding of reliability issues that may result from asking donors to self-report condom use, as well as a better understanding of factors underpinning the effectiveness of consistent condom use, such condom use errors and problems [27]. Our findings are consistent with and strongly supported by other research on MSM deferral policies across literature that encourage a deferral policy that is individual risk-based and applies to all donors regardless of sexual orientation or gender identity [4, 8, 10, 12, 13]. Finally, this policy shift should be paired with blood operators’ efforts to repair relationship with GBMSM communities – an effort that would likely impact community perception of these organizations and perhaps increase GBMSM’s interest in donating blood and willingness to donate blood.

## Data Availability

The datasets generated and/or analysed during the current study are not publicly available due to privacy or ethical restrictions. Please contact the corresponding author for further information.

## Abbreviations

CBS: Canadian Blood Services
GBMSM: Gay, bisexual, and other men who have sex with men
MSM: Men who have sex with men
NAT: Nucleic acid testing
PEP: Post-exposure prophylaxis
PrEP: Pre-exposure prophylaxis

## References

[1] Canadian Blood Services. MSM Research Program: Knowledge Synthesis Interim Report. 2020. https://www.blood.ca/en/blood/ami-eligible/men-who-have-sex-men/knowledge-synthesis-interim-report. Accessed 25 Nov 2021.

[2] Wainberg MA, Shuldiner T, Dahl K, Gilmore N (2010). Reconsidering the lifetime deferral of blood donation by men who have sex with men. Can Med Assoc J.

[3] Canadian Blood Services. Blood safety. https://www.blood.ca/en/blood/blood-safety (n.d.). Accessed 25 Nov 2021.

[4] Goldman M, Shih A W-Y, O’Brien SF, Devine D (2018). Donor deferral policies for men who have sex with men: past, present and future. Vox Sang.

[5] Skelly AN, Kolla L, Tamburro MK, Bar KJ (2020). Science over stigma: the need for evidence-based blood donation policies for men who have sex with men in the USA. Lancet Haemat.

[6] Park C, Gellman C, O’Brien M, Eidelberg A, Subudhi I, Gorodetsky EF (2021). Blood donation and COVID-19: reconsidering the 3-month deferral policy for gay, bisexual, transgender, and other men who have sex with men. Am J Public Health.

[7] Valentine, K. Blood donation in Australia: altruism and exclusion. In: Charbonneau J, Smith A, editors. Giving blood: the institutional making of altruism. New York Routledge; 2016. p. 183–189.

[8] Caruso J, Germain M, Godin G, Myhal G, Pronovost F, Morin M (2019). ‘One step closer’: Acceptability of a programme of plasma donation for fractionation from men who have sex with men. Vox Sang.

[9] Community-Based Research Centre. Sex Now Survey results reveal prevalence of change effort. 2020. https://www.cbrc.net/sex_now_survey_results_reveal_prevalence_of_change_efforts. Accessed Nov 25 2021.

[10] Grace D, Gaspar M, Klassen B, Lessard D, Brennan DJ, Lachowsky NJ (2020). It’s in me to give: Canadian gay, bisexual, and queer men’s willingness to donate blood if eligible despite feelings of policy discrimination. Qual Health Res.

[11] O’Brien SF, Goldman M, Robillard P, Osmond L, Myhal G, Roy É (2021). Donor screening question alternatives to men who have sex with men time deferral: Potential impact on donor deferral and discomfort. Transfusion.

[12] Grace D, Gaspar M, Lessard D, Klassen B, Brennan DJ, Adam BD (2019). Gay and bisexual men’s views on reforming blood donation policy in Canada: a qualitative study. BMC Public Health.

[13] Zahner GJ (2020). We Need Blood and I Am Not Allowed to Help. JAMA Intern Med.

[14] Brennan, DJ, Kesler, M, Lachowsky, NJ, Davies, A, Georgievski, G, Adam, B, et al. Sociodemographic and psychological predictors of seeking health information online among GB2M in Ontario: Findings from the #iCruise Project. International Journal of Sexual Health. 2021 Dec 16. 10.1080/19317611.2021.2000087. 10.1080/19317611.2021.2000087 PMC10903557 38596527

[15] Baggaley RF, White RG, Boily M-C (2008). Systematic review of orogenital HIV-1 transmission probabilities. Int J Epidemiol.

[16] Rothenberg RB, Scarlett M, del Rio C, Reznik D, O’Daniels C (1998). Oral transmission of HIV. AIDS.

[17] Vitinghoff E, Douglas J, Judon F, McKiman D, MacQueen K, Buchinder SP (1999). Per-contact risk of human immunodificiency virus tramnsmision between male sexual partners. Am J Epidemiol.

[18] del Romero J, Marincovich B, Castilla J, García S, Campo J, Hernando V (2002). Evaluating the risk of HIV transmission through unprotected orogenital sex. AIDS.

[19] Patel P, Borkowf CB, Brooks JT, Lasry A, Lansky A, Mermin J (2014). Estimating per-act HIV transmission risk: a systematic review. AIDS.

[20] Smith DK, Herbst JH, Xinjiang Z, Rose CE (2015). Condom effectiveness for HIV prevention by consistency of use among men who have sex with men in the United States. J Acquir Immune Defic Syndr.

[21] Jin F, Prestage GP, Imrie J, Kippax SC, Donovan B, Templeton DJ (2010). Anal sexually transmitted infections and risk of hiv infection in homosexual men. J Acquir Immune Defic Syndr.

[22] Jin F, Jansson J, Law M, Prestage GP, Zablotska I, Imrie JCG (2010). Per-contact probability of HIV transmission in homosexual men in Sydney in the era of HAART. AIDS.

[23] Canadian Blood Services. Donor questionnaire for blood, platelets and plasma. https://www.blood.ca/en/blood/donating-blood/donor-questionnaire (n.d.). Accessed 2 Apr 2022.

[24] Canadian Blood Services. ABCs of eligibility to donating blood, platelets and plasma. https://www.blood.ca/en/blood/am-i-eligible-donate-blood/abcs-eligibility (n.d.). Accessed 2 Apr 2022.

[25] Berg BL, Lune H (2012). Qualitative research methods for the social sciences.

[26] Adam BD, Globerman J, Elliott R, Corriveau P, English K, Rourke S (2016). HIV Positive People’s Perspectives on Canadian Criminal Law and Non-Disclosure. Canadian Journal of Law and Society.

[27] Sanders SA, Yarber WL, Kaufman EL, Crosby RA, Graham CA, Milhausen RR (2012). Condom use errors and problems: a global view. Sexual health.

[28] Romero-Domínguez L, Martín-Santana JD, Sánchez-Medina AJ, Beerli-Palacio A. Blood donation barriers: How does donor profile affect them? International Review on Public and Nonprofit Marketing. 2021. 10.1007/s12208-021-00303-5.

[29] Schreiber GB, Schlumpf KS, Glynn SA, Wright DJ, Tu Y, King MR (2006). Convenience, the bane of our existence, and other barriers to donating. Transfusion.

[30] Guglielmetti Mugion R, Pasca MG, Di Di PL, Renzi MF (2021). Promoting the propensity for blood donation through the understanding of its determinants. BMC Health Serv Res.

[31] Haire B, Whitford K, Kaldor JM (2018). Blood donor deferral for men who have sex with men: still room to move. Transfusion.

[32] Grace D, Gaspar M, Klassen B, Lessard D, Anand P, Brennan DJ (2021). Stepping Stones or Second Class Donors?: a qualitative analysis of gay, bisexual, and queer men’s perspectives on plasma donation policy in Canada. BMC Public Health.

[33] Haire BG, Kaldor JM (2019). Prevalence of Transfusion-transmissible Infections, Not “Infection Pressure”, Should Dictate Suitability to Donate Blood. Clin Infect Dis.

[34] Rodger AJ, Cambiano V, Bruun T, Vernazza P, Collins S, van Lunzen J (2016). Sexual Activity Without Condoms and Risk of HIV Transmission in Serodifferent Couples When the HIV-Positive Partner Is Using Suppressive Antiretroviral Therapy. JAMA.

[35] Canadian Blood Services. Evolving eligibility criteria for gay, bisexual and other men who have sex with men. https://www.blood.ca/en/blood/am-i-eligible-donate-blood/men-who-have-sex-men/eligibility-criteria-gay-bisexual-and-other (n.d.). Accessed 16 Dec 2021.

[36] NHS Blood Donation. Men who have sex with men. NHS Blood Donation. https://www.blood.co.uk/who-can-give-blood/men-who-have-sex-with-men/ (n.d.). Accessed 2 Apr 2022.

